# A combination of cellular biomarkers predicts failure to respond to rituximab in rheumatoid arthritis: a 24-week observational study

**DOI:** 10.1186/s13075-016-1091-1

**Published:** 2016-08-24

**Authors:** Martin H. Stradner, Christian Dejaco, Kerstin Brickmann, Winfried B. Graninger, Hans Peter Brezinschek

**Affiliations:** Division of Rheumatology and Immunology, Medical University of Graz, Graz, Austria

**Keywords:** Rheumatoid arthritis, Biomarker, Rituximab, T cells, Plasmablasts, Lymphocytes

## Abstract

**Background:**

Although B cell depletion with rituximab (RTX) is an effective treatment strategy in rheumatoid arthritis (RA), one third of patients do not achieve remission or low disease activity (LDA). Thus, identifying patients who will benefit from RTX is highly desirable. In the present study we investigated whether lymphocyte subsets other than B cells are predictors of a clinical response to RTX treatment.

**Methods:**

Patients with RA who were receiving RTX for the first time were included in an observatory registry. Clinical assessments, complete blood count and flow cytometry of lymphocyte subsets were obtained at baseline and at week 24 after RTX. Complete data were available for 44 patients. Logistic regression and receiver operating characteristic curve analyses were computed to analyze the predictive value of lymphocyte subsets for European League Against Rheumatism (EULAR) response and LDA (defined as disease activity score in 28 joints (DAS28) ≤3.2) at week 24.

**Results:**

EULAR responders had lower total lymphocyte counts (LC), T cells and CD4 + T cells at baseline. Although these parameters were independent predictors of EULAR response they failed in determining who would reach LDA. In contrast, LC >2910/μl or plasmablast frequency >2.85 % at baseline predicted a significantly higher DAS28 at week 24 after RTX and identified patients not achieving LDA at week 24 with sensitivity of 93.3 % and specificity of 44.8 %.

**Conclusions:**

A combination of LC and plasmablast frequency identifies patients with RA who will not benefit from RTX with high probability.

**Electronic supplementary material:**

The online version of this article (doi:10.1186/s13075-016-1091-1) contains supplementary material, which is available to authorized users.

## Background

With the introduction of biologic disease-modifying anti-rheumatic drugs (DMARDs) (bDMRDs) the armamentarium to fight rheumatoid arthritis (RA) has been dramatically enlarged [1]. However, we are unable to predict which of these therapies would be optimal for a certain patient. For example, B cell depletion with the chimeric monoclonal antibody rituximab (RTX) is an effective treatment strategy for RA. However, a considerable proportion of around 30 % of patients with RA treated with RTX fail to respond, particularly after previous therapy with tumor necrosis factor (TNF)-α inhibitors [2]. Identification of patients likely to respond to RTX treatment would result in an optimized treatment strategy reducing unnecessary socio-economic costs and potential side effects. Currently available clinical and laboratory parameters predicting the success of RTX therapy include the presence of rheumatoid factor (RF) and/or anti-citrullinated peptide antibodies (ACPA), and the absence of current glucocorticoid therapy [3–5]. In addition, high serum calprotectin has been associated with good or moderate response to RTX [6]. Furthermore, several authors have demonstrated that patients with RA who have a high frequency of plasmablasts are less likely to respond to RTX [7–11].

All these factors, however, have been established to predict EULAR response. Current recommendations for the treatment of RA define remission or low disease activity (LDA) in patients with long-standing disease as the goal of treatment after 6 months [12], a target that is not achieved with a moderate EULAR response in many cases.

Whether factors other than B cell subsets or their products might help us to find the optimal therapy for a particular patient is still unknown. As several immune competent cells are involved in the pathogenesis of RA [13] the efficacy of a certain bDMARD might be determined not only by its defined target but also by cells or molecules interacting with it. Thus, the influence of RTX on T cells became an alternative focus of recent investigations [14–16].

In the present study, we analyzed 1) whether baseline levels of lymphocyte subsets other than those of B cells may predict clinical response to RTX and 2) whether changes in T cell subsets correlate with clinical outcomes.

## Methods

### Patients

Data from the Austrian Rituximab registry were used for this study. The clinical protocol with a detailed description of inclusion and exclusion criteria, interventions, and clinical and laboratory assessments has been described earlier [11]. Briefly, consecutive patients were included who fulfilled the 1987 American College of Rheumatology (ACR) classification criteria for RA [17] and were receiving RTX for the first time. All patients received 1000 mg RTX preceded by the administration of 100 mg of prednisolone at baseline and after 2 weeks. Complete blood count, lymphocyte analysis, and assessment of disease activity score in 28 joints (DAS28) using the erythrocyte sedimentation rate were carried out before RTX treatment and at week 24. Patients were classified according to European League Against Rheumatism (EULAR) as good responders, moderate responders, or non-responders [18]. LDA was defined as a DAS28 ≤ 3.2 [19].

### Lymphocyte analysis

Blood cell counts in peripheral blood samples were obtained using a Beckman Coulter HMX hematology analyzer (Beckman Coulter, Miami, FL, USA). For determination of lymphocyte subsets whole blood was stained for CD45, CD3, CD19, CD4, CD8, CD56, and CD16 using the BD Multitest IMK kit (Becton Dickinson, Heidelberg, Germany). After fixation and erythrocyte lysis according to the manufacturer’s protocol, cells were analyzed on a FACS Calibur flow cytometer (Becton Dickinson) using FACS Diva software (Becton Dickinson).

### Statistical analysis

All statistical analyses were performed using the SPSS program, version 21.0 (IBM, Chicago, IL, USA). Figures were generated using GraphPad Prism 5 (La Jolla, CA, USA). The Kolmogorov-Smirnov test was used to check the normality of metric data. Two-group comparisons were performed using the unpaired *t* test (parametric data) or Mann-Whitney *U* test (non-parametric data). For pairwise analysis of non-normally distributed data we used Wilcoxon matched pairs test. Multiple comparisons were calculated using one-way analysis of variance (ANOVA) (parametric data) or the Kruskal-Wallis test (non-parametric data) and the appropriate post hoc tests (the Bonferroni and Mann-Whitney *U* test, respectively). We conducted inclusive logistic regression analyses (maximum likelihood method) to investigate the association between EULAR response or DAS28 ≤ 3.2 after RTX treatment (dependent variable) and leucocyte count (LC), B cell, T cell, CD4^+^T cell, CD8^+^T cell, and natural killer (NK) cell count, and a combined LC and plasmablast score (predictors of primary interest) adjusting for DAS28, current glucocorticoid therapy, disease duration, sex, failure to respond to more than one TNF inhibitor and ACPA status. To identify possible cutoffs for baseline LC and plasmablast frequency distinguishing between EULAR responders and non-responders, receiving operating characteristic (ROC) curves were constructed by plotting sensitivity against one minus specificity varying the cutoffs and calculating the area under the curve (AUC). Data are presented as mean ± standard error of the mean unless indicated otherwise. Odds ratios (OR) are presented with the 95 % confidence interval (CI) in brackets.

## Results

### Baseline characteristics

Because of technical problems, complete data on T and B cell cytometry was available for only 44 of the 52 patients with RA who were receiving RTX. At week 24, there were 33 patients (75.0 %) classified as EULAR responders and 15 patients (34.1 %) had achieved remission or LDA. Baseline characteristics are summarized in Table 1. The baseline characteristics are representative of a typical RA cohort with long-standing RA and high disease activity.

**Table 1:** Baseline characteristics of patients included in the study

Parameter	Non-responders	Responders	*P* value	DAS28 > 3.2 (week 24)	DAS28 ≤ 3.2 (week 24)	*P* value
	(n = 11)	(n = 33)		(n = 29)	(n = 15)	
Age in years (mean ± SD)	58.9 ± 10.8	62.5 ± 13.3	0.423	61.8 ± 13.8	61.3 ± 10.7	0.898
Female gender, %	72	81	0.640	76	87	0.411
Disease duration in years (mean ± SD)	15.1 ± 12.7	12.9 ± 8.8	0.536	12.7 ± 10.5	15.4 ± 8.1	0.428
DAS28-ESR (mean ± SD)	5.0 ± 1.1	6.0 ± 1.1	**0.008**	5.8 ± 1.1	5.7 ± 1.3	0.753
RF-positive, %	100	87	0.236	86	100	0.138
ACPA-positive, %	78	76	0.909	72	85	0.399
Corticosteroids, %	36	30	0.061	34	26	0.608
Previous TNF inhibitors (mean ± SD)	1.7 ± 1.2	1.2 ± 0.9	0.194	1.5 ± 1.1	1.2 ± 0.7	0.346

Significant *P* values are indicated in bold numbers

DAS28 disease activity score in 28 joints, *SD* standard deviation, *ESR* erythrocyte sedimentation rate, *RF* rheumatoid factor IgM, *ACPA* anti-citrullinated peptide antibody, *TNF* tumor necrosis factor

### Baseline lymphocyte counts predict EULAR response but not LDA

**Fig. 1:**
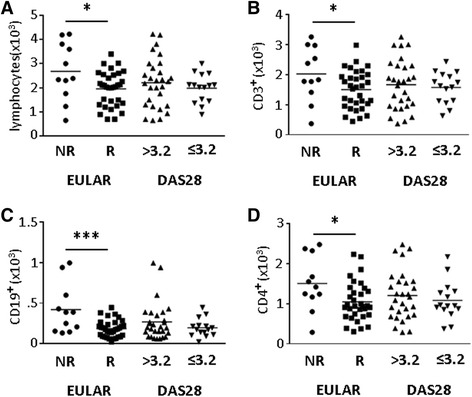
Rituximab (RTX) non-responders (*NR*) have higher baseline lymphocyte, T cell, and CD4^+^lymphocyte counts than responders (*R*). Lymphocyte (**a**), CD45^+^CD3^+^ (**b**), CD45^+^CD19^+^ (**c**), and CD45^+^CD3^+^CD4^+^ (**d**) counts were obtained before treatment with RTX. European League Against Rheumatism (*EULAR*) response and disease activity score in 28 joints (*DAS28*) were assessed at week 24 after treatment with RTX. Data are presented as cells/μl. Each *dot* represents a patient; *horizontal bars* indicate the mean. Statistical significance was assessed using the Mann-Whitney *U* test. *P <0.05, ***P <0.001

We analyzed whether baseline levels of lymphocyte subsets differed between responders and non-responders to RTX therapy. Interestingly, baseline total lymphocyte count (LC) was significantly higher in the non-responder group compared to patients with EULAR response, as depicted in Fig. 1a (2681 ± 360/μl and 1956 ± 124/μl, respectively, *P* = 0.019). We found no significant difference in baseline LC between patients with DAS28 > 3.2 and DAS28 ≤ 3.2 at week 24.

Next we investigated if one or more of the lymphocyte subsets were responsible for the negative correlation between EULAR response and cell count. We observed a higher CD3^+^ T-cell count in non-responders than in responders (2022 ± 904/μl, 1501 ± 110/μl, respectively, *P* = 0.040, Fig. 1b). In addition, non-responders had higher total numbers of B cells compared to responders (421 ± 92/μl and 185 ± 18/μl, respectively, *P* = 0.001, Fig. 1c). A detailed analysis of the B cell subsets in our cohort has already been published [11]. Among the T-cell subsets we found higher levels of CD4^+^T cells in the non-responder group (1504 ± 205/μl and 1047 ± 84/μl, respectively, *P* = 0.19; Fig. 1d). Importantly, at baseline none of these populations was significantly different between patients with a DAS28 > 3.2 or a DAS28 ≤ 3.2 at week 24. This was also true for the plasmablast frequency, a predictor of EULAR response identified previously [11]. Furthermore, we did not detect any significant difference in the prevalence of CD8^+^T cells and natural killer cells (data not shown). In addition, DMARD therapy and glucocorticoid use had no influence on lymphocyte counts or lymphocyte subsets (Additional file 1: Table S1). In univariate logistic regression analysis high baseline LC, B cell, T cell, and CD4^+^T cell counts were negative predictors of EULAR response but there were no predictors of LDA (Table 2).

**Table 2:** Univariate logistic regression models predicting EULAR response and DAS28 ≤ 3.2 at week 24 after rituximab

Baseline variables	EULAR response		DAS28 ≤ 3.2	
	OR (95 % CI)	*P* value	OR (95 % CI)	*P* value
Leucocytes^a^	1.00 (1.00–1.00)	0. 926	1.00 (1.00–1.00)	0.892
LC^a^	0.91 (0.83–0.99)	**0.030**	0.97 (0.90–1.04)	0.420
Natural killer cells^a^	0.93 (0.59–1.48)	0.766	0.85 (0.50–1.41)	0.524
B cells^a^	0.48 (0.26–0.87)	**0.015**	0.79 (0.52–1.20)	0.274
T cells^a^	0.90 (0.81–1.00)	**0.050**	0.98 (0.91–1.07)	0.692
CD4^+ a^	0.87 (0.76–0.99)	**0.029**	0.96 (0.86–1.08)	0.491
CD8^+ a^	0.94 (0.74–1.20)	0.607	1.061 (0.85–1.33)	0.640

^a^Absolute values, multiplied by 100. *EULAR* European League Against Rheumatism, *DAS28* disease activity score in 28 joints, *CI* confidence interval, *LC* total lymphocyte count, *OR* odds ratio (indicated per 100 cells/μl increase)

Significant *P* values are indicated in bold numbers

Multivariate regression analysis as described in Methods was then carried out to adjust the data for possible confounders, confirming the results from univariate analysis: LC, T cell, B cell, and CD4^+^T cell counts were independent negative predictors of EULAR response (Table 3).

**Table 3:** Multivariate logistic regression models predicting EULAR response at week 24 after rituximab

Baseline variables	EULAR response	
	OR (95 % CI)	*P* value
LC^a^	0.88 (0.80–0.89)	0.021
B cells^a^	0.43 (0.22–0.88)	0.020
T cells^a^	0.87 (0.70–0.96)	0.018
CD4^+ a^	0.82 (0.70–0.97)	0.015

^a^Absolute values, multiplied by 100. *EULAR* European League Against Rheumatism,*CI* confidence interval, *LC* total lymphocyte count, *OR* odds ratio (indicated per 100 cells/μl increase)

### Combining baseline LC and plasmablast frequency predicts low disease activity

**Fig. 2:**
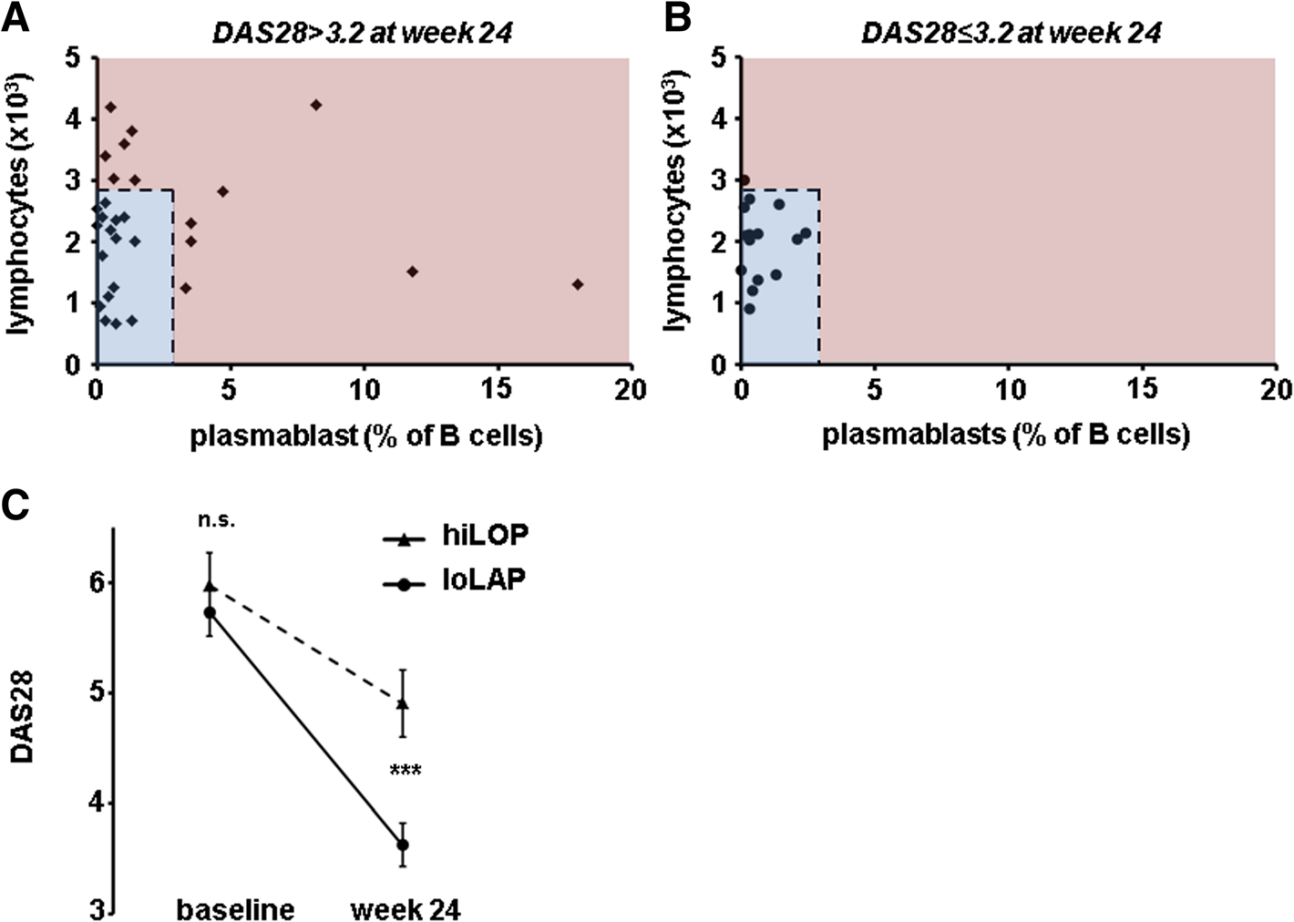
Patients with high total lymphocyte counts (LC) or high frequency of plasmablasts (*hiLOP*) have higher disease activity. Baseline values of LC and plasmablasts in patients not achieving low disease activity (LDA) after 24 weeks (**a**). Baseline values of LC and plasmablasts in patients achieving LDA after 24 weeks (**b**). *Dotted lines* indicate the cutoff for baseline. *Red* and *blue* indicate the area for baseline hiLOP or low number of total lymphocyte counts and plasmablasts (*loLAP*), respectively. Change in disease activity score in 28 joints (*DAS28*) in patients with baseline loLAP and hiLOP (**c**). Statistical significance was assessed using the Mann-Whitney *U* test; ***P <0.001

The current focus in RA therapy is to achieve remission or LDA by 6 months [12]. None of the cellular biomarkers evaluated so far solely predicted LDA. Therefore, we investigated whether a combination of two biomarkers could be used for predicting LDA. We hypothesized that these biomarkers would have to identify a different patient group each, in order to improve the prediction. By blotting each of the candidate biomarkers against each other, we found that high LC and high plasmablasts, a predictor of EULAR response identified previously [11], each recognized a different population of patients not reaching LDA by week 24 (Fig. 2a). In contrast, all patients who did reach LDA had low LC and low plasmablast frequency (Fig. 2b). This finding prompted us to use a combination of both biomarkers in inclusive disjunction. The cutoffs for both values were chosen to yield at least 90 % specificity for non-response generated by ROC analysis (Additional file 2: Figure S1). Patients with elevated LC >2910/μl *or* plasmablast frequency >2.85 % (hiLOP) at baseline had a significantly higher DAS28 at week 24 after treatment with RTX than patients who had LC and plasmablast frequency below the thresholds (loLAP) (DAS28 of 4.9 ± 0.3 and 3.7 ± 0.2, respectively; *P* = 0.002. Fig. 2c). Positive and negative predictive values for achieving a DAS28 ≤ 3.2 are given in Table 4.

**Table 4:** Contingency table of loLAP or hiLOP scores and disease activity at week 24

	loLAP	hiLOP	
DAS28 ≤ 3.2	14	1	Sensitivity: 93.3 %
DAS28 > 3.2	16	13	Specificity: 44.8 %
	PPV: 46.7 %	NPV: 92.9 %	

*hiLOP* high number of total lymphocyte count or plasmablasts, *loLAP* low number of total lymphocyte count and plasmablasts, *DAS28* disease activity score in 28 joints; *PPV* positive predictive value, *NPV* negative predictive value

To test if hiLOP was an independent predictor of a DAS28 ≤ 3.2 we performed multivariate regression corrected for possible confounders. Indeed, baseline hiLOP significantly and independently predicted that patients treated with RTX would not reach DAS28 ≤ 3.2 at week 24 (OR 0.11, 95 % CI 0.01–0.89, *P* = 0.037). In contrast, other combinations of LC, plasmablast frequency, T cell, B cell, or CD4^+^T cell counts were not predictive of a DAS28 ≤ 3.2 in our analysis (data not shown).

### T cell numbers are reduced after treatment with RTX but do not correlate with clinical response

**Fig. 3:**
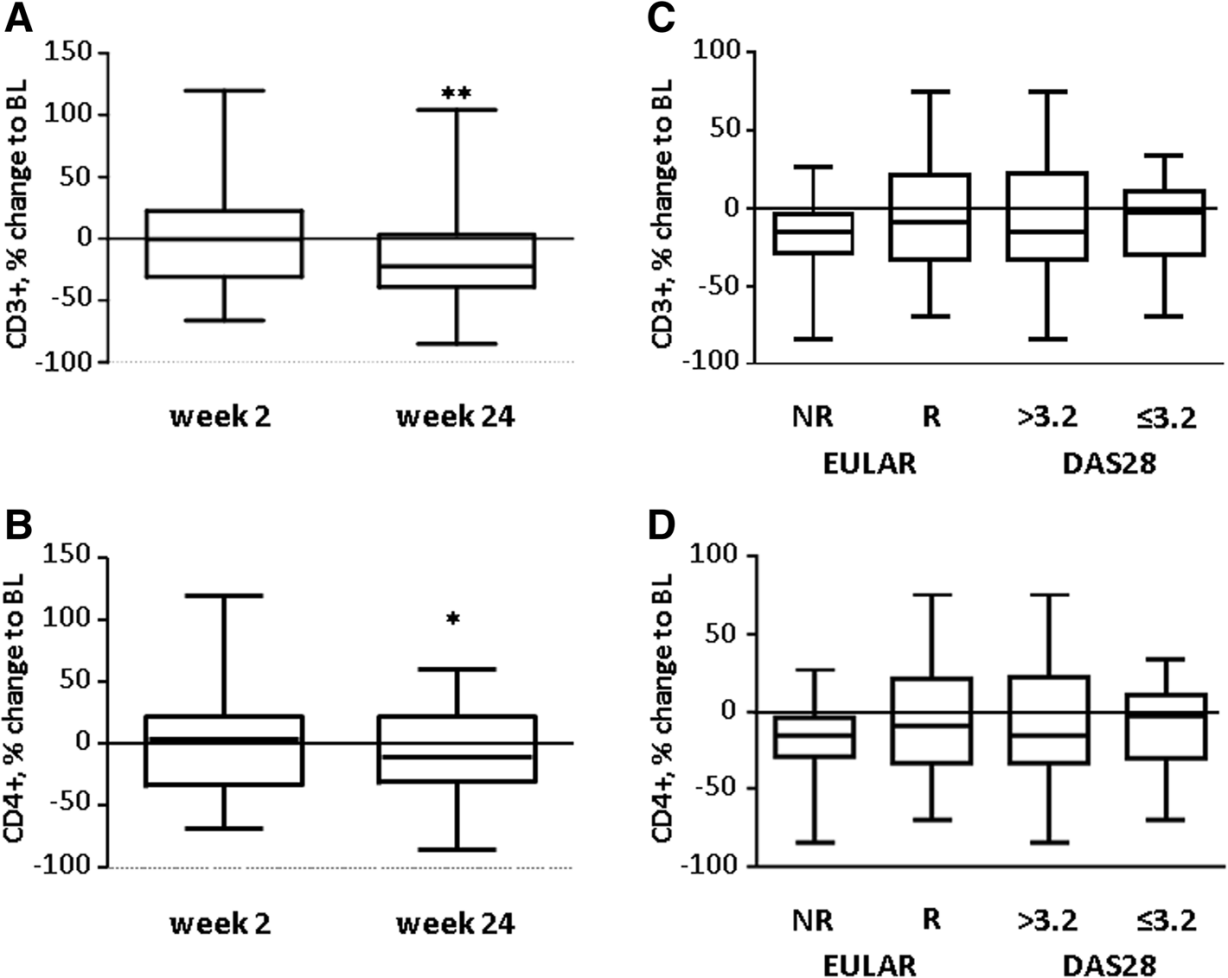
T cell numbers are reduced after treatment with rituximab (RTX) but do not correlate with clinical response. CD45^+^CD3^+^ (**a**, **c**) and CD45^+^CD3^+^CD4^+^ (**b**, **d**) lymphocytes of all patients were analyzed at the indicated time points after treatment with RTX (**a**, **b**) or at week 24, separated according to European League Against Rheumatism (*EULAR*) response and disease activity in 28 joints (*DAS28*) at week 24 (**c**, **d**). Data are presented as percentage change of the indicated lymphocyte subset compared to baseline (*BL*). *Boxes* represent the median, with 50 % of cases within the boxes. *Whiskers* represent the minimum and maximum. Statistical significance was assessed using the Wilcoxon matched pairs test (**a**, **b**) and one-way analysis of variance (**c**, **d**); *P <0.05, **P <0.01. *R* responders, *NR* non-responders

We also tested whether a change in the absolute T cell count after therapy with RTX correlates with EULAR response as reported previously [14, 20]. The absolute number of T lymphocytes at week 24 after RTX was reduced to 79.9 % of baseline levels (1652 ± 113/μl to 1320 ± 88/μl, *P* = 0.003, Fig. 3a). We also found a reduction of CD4^+^T lymphocytes (1174 ± 88/μl to 933 ± 60/μl, *P* = 0.007, Fig. 3b), CD8^+^T lymphocytes (492 ± 44/μl to 399 ± 45/μl, *P* = 0.001) but not natural killer cell numbers (242 ± 24/μl to 229 ± 24/μl, *P* = 0.475) by flow cytometry. However, neither the reduction of T lymphocytes nor the decrement of CD4^+^ T-cell levels was associated with a EULAR response or a DAS28 ≤ 3.2 as outlined in Fig. 3c and d (*P* > 0.05). Changes within the CD8^+^T lymphocyte subset were also similar in responders and non-responders (data not shown).

## Discussion

Selection of a new DMARD for patients with RA who are responding insufficiently to a current therapy is a delicate decision. Currently the best predictor of reaching remission or LDA is not an individual biomarker, but the level of disease activity and its reduction, especially during the first 3 months of treatment [21].

Measurement of the LC is simple and cheap. In our study 91 % of responders had LC below the cutoff of <2910 lymphocytes/μl. Interestingly, this value is higher than that reported by Ferraccioli et al. [3], who found that a lymphocyte count below 1875/μl is an excellent predictor of a good EULAR response following B cell depletion. The difference between cutoff values may be related to differences in the two RA cohorts: our patients had higher disease activity (5.8 versus 4.6), fewer of them took glucocorticoids (30 % versus 80 %), and all were RF-IgM-positive (100 % versus 85 %).

As LC characterized a different population of non-responders compared to high levels of plasmablasts, we combined the baseline values of LC and plasmablast frequency and generated an LOP score that has high negative predictive value and sensitivity to identify patients who will not achieve low disease activity under RTX. This is especially important for treatment decisions in clinical practice, where the target of treatment is remission or at least low disease activity [12]. Our data suggest that LC and B cell subtyping should be performed in patients before treatment decisions. Treatment options other than RTX may be preferred for patients who have hiLOP.

We observed a decrease in T cell numbers 24 weeks after treatment with RTX. In line with our findings, in two recent studies there was also a reduction in circulating T cells in patients treated with RTX [14, 20]. Intriguingly, the reduction in overall T cells after RTX in these studies correlated with EULAR response, which we did not confirm in our cohort. Again, differences in patient characteristics may account for the divergent findings, as more of our patients were RF-positive and fewer were taking steroids.

How RTX might affect T cell homeostasis is still under discussion. One possibility could be a direct effect on CD20^+^ T cells. This has recently been demonstrated in patients with multiple sclerosis [22], but it seems unlikely in RA because of the small number of peripheral T cells expressing this molecule as Melet et al. have indicated [14]. Evidence for an indirect effect of RTX comes from observations in lupus-prone MRL-*lpr/lpr* mice. Analysis of B-cell-intact and B-cell-deficient mice demonstrates that the expansion of activated and memory T cells is highly dependent on the presence of B cells [23]. Furthermore, B cell depletion limits the generation of CD4+ memory T cells and reduces protection against disseminating virus infection [24]. Thus, diminishing the number of B cells reduces signals for the activation and expansion of T cells. In line with this are reports of a diminished number of activated CD69^+^ or CD154^+^ T cells after successful B cell depletion therapy in humans with systemic lupus erythematosus [25, 26].

How low numbers of total lymphocytes may be beneficial for patients with RA treated with RTX is unknown. Approximately 75 % of total lymphocytes are T cells and 60 % of these are CD4^+^ [27]. Within this population are helper cells that are able to promote the survival of autoreactive B cells [28]. Starting with a low number of T lymphocytes the additional decrease in these cells after RTX might be sufficient to disrupt the reciprocal activation of auto-reactive T and B cells.

A limitation of our study is the small sample size and the lack of detailed characterization of T cells. In addition, as all our patients were RF-positive it was not possible to meaningfully assess any statistical association between autoantibodies and EULAR response. ACPA status, however, was neither linked with EULAR response nor confounded the predictive values of LC, CD4^+^T lymphocyte count, or hiLOP outcome.

Whether patients with high LC or plasmablast frequency would benefit from an additional cycle of rituximab as suggested by Vital et al. [8] cannot be answered by our study. As a first step we would like to confirm our data in a prospective clinical trial selecting patients with RA for RTX therapy according to their LOP score.

## Conclusions

Currently, there are several bDMARDs available for the treatment of RA, but despite a plethora of data from different studies no single biomarker has emerged that might predict the response to different therapies. Here we describe a combination of biomarkers, i.e., high LC and plasmablast frequency at baseline, that reliably identify patients who are likely to fail to respond to RTX treatment.

## Additional files

Additional file 1: Table S1. One-way ANOVA for leucocyte subsets and treatment. (DOCX 17 kb) Additional file 2: Figure S1. ROC curves of LC and CD4^+^lymphocyte counts. Sensitivity for EULAR response blotted against false negative rate (1-specificity), at various threshold settings for baseline LC and CD45^+^CD3^+^CD4^+^ counts. The increasing area under the curve (AUC) corresponds to higher diagnostic accuracy. (TIFF 42 kb)

## Abbreviations

ACPA: anti-citrullinated peptide antibodies
ACR: American College of Rheumatology
ANOVA: analysis of variance
bDMRD: biologic disease-modifying anti-rheumatic drug
BL: baseline
DAS28: disease activity score 28
EULAR: European League Against Rheumatism
hiLOP: high number of total lymphocyte count or plasmablasts
LC: total lymphocyte counts
LDA: low disease activity
loLAP: low number of total lymphocyte count and plasmablasts
RA: rheumatoid arthritis
RF: rheumatoid factor
RTX: rituximab
TNF: tumor necrosis factor
