# Supplementary material for: A combination of cellular biomarkers predicts failure to respond to rituximab in rheumatoid arthritis: a 24-week observational study
Source: Arthritis Res Ther. 2016 Aug 24;18(1):190. doi: 10.1186/s13075-016-1091-1 (PMC4997751; doi:10.1186/s13075-016-1091-1)
Supplement: Additional file 1: Table S1. — One-way ANOVA for leucocyte subsets and treatment. (DOCX 17 kb) [file 13075_2016_1091_MOESM1_ESM.docx]

| **Table S1: One-way ANOVA for leucocyte subsets and treatment** | | | |
| --- | --- | --- | --- |
| **Leukocyte population** | **Treatment** | **Mean (±SEM)** | ***P*-value** |
| **LC** | prednisolone<5mg | 2128.9 (±335.6) | *0.75* |
|  | prednisolone>5mg | 2760.0 (±640.0) |  |
|  | methotrexate | 2007.3 (±221.8) |  |
|  | other | 2127.7 (±167.9) |  |
| **T cells** | prednisolone<5mg | 1670.3 (±242.4) | *0.53* |
|  | prednisolone>5mg | 2368.5 (±608.5) |  |
|  | methotrexate | 1535.7 (±171.8) |  |
|  | other | 1801.3 (±577.7) |  |
| **CD4+** | prednisolone<5mg | 1177.1 (±177.6) | *0.81* |
|  | prednisolone>5mg | 1599.5 (±631.5) |  |
|  | methotrexate | 1154.9 (±138.9) |  |
|  | other | 1179.7 (±460.5) |  |
| **B cells** | prednisolone<5mg | 246.7 (±94.2) | *0.93* |
|  | prednisolone>5mg | 147.0 (±8.0) |  |
|  | methotrexate | 219.5 (±39.3) |  |
|  | other | 228.8 (±66.0) |  |
| **plasmablasts** | prednisolone<5mg | 2.0 (±1.2) | *0.61* |
|  | prednisolone>5mg | 0.5 (±0.2) |  |
|  | methotrexate | 0.9 (±0.3) |  |
|  | other | 0.9 (±0.5) |  |
| Patients were grouped according to treatment at baseline and analyzed for differences in leucocyte populations. *LC* total lymphocyte count; *other* leflunomide and chloroquine | | | |
