# Supplementary figures and images for: A combination of cellular biomarkers predicts failure to respond to rituximab in rheumatoid arthritis: a 24-week observational study
Source: Arthritis Res Ther. 2016 Aug 24;18(1):190. doi: 10.1186/s13075-016-1091-1 (PMC4997751; doi:10.1186/s13075-016-1091-1)

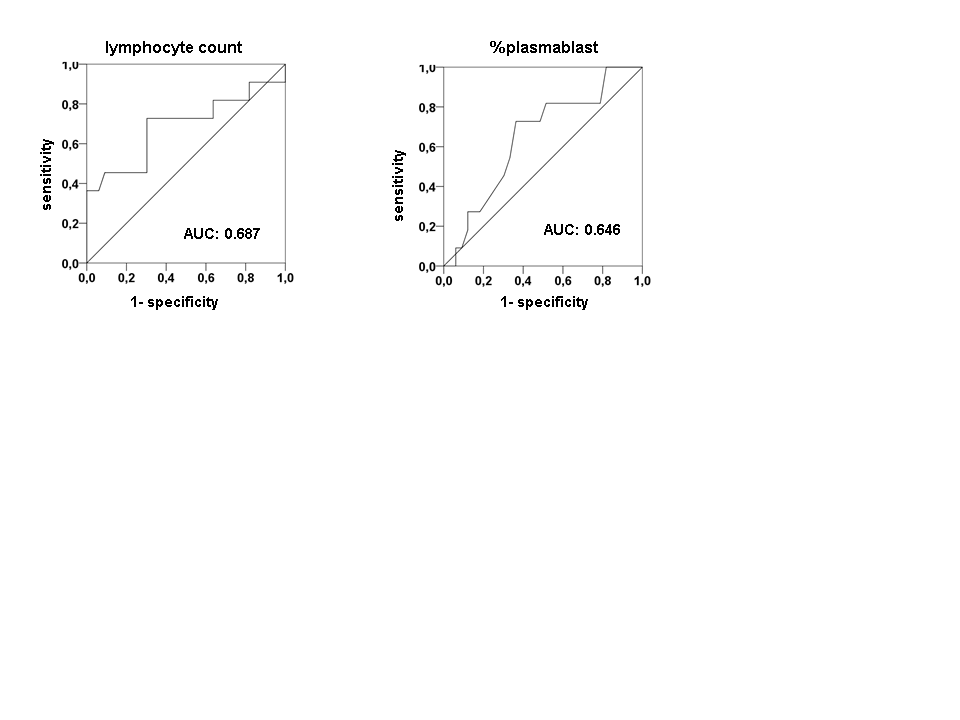

Supplement: Additional file 2: Figure S1. — ROC curves of LC and CD4+lymphocyte counts. Sensitivity for EULAR response blotted against false negative rate (1-specificity), at various threshold settings for baseline LC and CD45+CD3+CD4+ counts. The increasing area under the curve (AUC) corresponds to higher diagnostic accuracy. (TIFF 42 kb) [file 13075_2016_1091_MOESM2_ESM.tiff]
